# Association of SNP Haplotypes of *HKT* Family Genes with Salt Tolerance in Indian Wild Rice Germplasm

**DOI:** 10.1186/s12284-016-0083-8

**Published:** 2016-03-29

**Authors:** Shefali Mishra, Balwant Singh, Kabita Panda, Bikram Pratap Singh, Nisha Singh, Pragati Misra, Vandna Rai, Nagendra Kumar Singh

**Affiliations:** National Research Centre on Plant Biotechnology, Pusa Campus, New Delhi, 110012 India; Jacob School of Biotechnology and Bioengineering, Sam Higginbottom Institute of Agriculture, Technology and Sciences, Allahabad, 211007 India

**Keywords:** Allele mining, HKT, Association, Salt stress, NaCl, Wild rice

## Abstract

**Background:**

Rice is one of the most important crops for global food security but its productivity is adversely affected by salt stress prevalent in about 30 % of the cultivated land. For developing salt-tolerant rice varieties through conventional breeding or biotechnological interventions, there is an urgent need to identify natural allelic variants that may confer salt tolerance. Here, 299 wild rice accessions collected from different agro-climatic regions of India were evaluated during growth under salt stress. Of these 95 representative accessions were sequenced for members of *HKT* ion transporter family genes by employing Ion Torrent PGM sequencing platform.

**Results:**

Haplotype analysis revealed haplotypes H5 and H1 of *HKT1*;*5* and *HKT2*;*3*, respectively associated with high salinity tolerance. This is the first study of allele mining of eight members of *HKT* gene family from Indian wild rice reporting a salt tolerant allele of HKT2;3. HKT1;5 also showed a salt tolerant allele from wild rice. Phylogenetic analysis based on the nucleotide sequences showed different grouping of the *HKT* family genes as compared to the prevailing protein sequence based classification.

**Conclusions:**

The salt tolerant alleles of the HKT genes from wild rice may be introgressed into modern high yielding cultivars to widen the existing gene pool and enhance rice production in the salt affected areas.

**Electronic supplementary material:**

The online version of this article (doi:10.1186/s12284-016-0083-8) contains supplementary material, which is available to authorized users.

## Background

Rice (*Oryza sativa* L.) is cultivated around the world and consumed by more than 50 % of the global human population (Mohanty 2013). The current status of rice production is 495.63 million tonnes (MT) which increased by only 9 MT in the preceding 4 year block (2011–2014) as compared to 80 MT increase in two such 4 year blocks during 2004–2011 (http://faostat3.fao.org/browse/Q/*/E). In contrast, the global population is projected to increase by 25 % to 9.2 billion by 2050 (Schroeder et al. 2013). Rice production must increase correspondingly by 70 % to fulfil the growing demand (FAOSTAT 2009). Fifteen percent of the land currently used for agricultural practices is at saturation point to maintain environmentally sustainable production, therefore innovations are needed to enhance crop productivity to meet the projected demand (Rockström et al. 2009). However, multiple abiotic stresses specifically salinity changes the soil texture and creates unfavourable conditions for rice production by severe inhibition of plant growth and development (Miller and Donahue 1995). Globally 45 Mha of irrigated and 32 Mha of rain fed agriculture are affected by salinity (http://www.fao.org/soils-portal/en/). Furthermore, irrigation with brackish water, tidal waves and tsunami continue to increase the soil salinity (Schroeder et al. 2013). According to one estimate up to 50 % of the present arable land may be affected by salinity by 2050 (Wang et al. 2003). Therefore, we need to explore natural genetic resources to find novel genes and alleles that can help withstand high salt concentration and maintain crop productivity.

Salinity is characterised by presence of exchangeable sodium ions (Na^+^) which imposes both ion toxicity and osmotic stress on the plant and alters the physiological status and ionic homeostasis of the cell. Crop plants are vulnerable to salinity and further their level of sensitivity and mechanism of tolerance depends on the growth stage of the plant (Hossain et al. 2015). Rice is most sensitive at the seedling stage and beyond 3 dSm^−1^ of electrical conductivity, seedlings are considered under stress and yield loss due to salinity is measured at twelve percent reduction per dSm^−1^ above threshold level (Lutts et al. 1995; Maas and Hoffman 1977). However, in most of the modern high yielding rice varieties, 50 % yield reduction occurs at 6 dSm^−1^ and they become totally unproductive beyond 12 dSm^−1^ (Linh et al. 2012). A limited number of large scale screening of germplasm for salinity stress has been conducted (Platten et al. 2013). Most of the genetic studies have focused on *Saltol* QTL derived from Indian rice landrace Pokkali which provides seedling stage salt tolerance (Thomson et al. 2010). There is very little work on the genetics of reproductive stage salt tolerance in rice (Mohammadi et al. 2013). The present understanding of salt tolerance mechanisms has facilitated exploitation of spatially located membrane transporters (Zhu 2001; Munns and Tester 2008). The known mechanisms include sequestration of ions in vacuole and exclusion of ions from root and leaves (Munns and Tester 2008). Thus, allelic variations in the sequence of ion transporter genes are likely to play a significant role in providing effective tolerance to salt stress.

In the present study we selected eight different transporters of the *HKT* gene family. These genes are further classified into two subfamilies based on their amino acid sequence similarity and differences in their Na^+^ and K^+^ selectivity (Horie et al. 2001), though product of each of these is a transmembrane protein (Horie et al. 2009; Mäser et al. 2001; Platten et al. 2006). Functionally, products of subfamily 1 [HKT1;1, HKT1;2, HKT1;3, HKT1;4 and HKT1;5] are Na + specific transporters and have S-G-G-G signature while products of subfamily 2 [HKT2;1, HKT2;2, HKT2;3 and HKT2;4] are Na^+^-K^+^ co-transporters or Na^+^-K^+^ uniporters with G-G-G-G signature (Jabnoune et al. 2009; Mäser et al. 2002). Oomen et al. 2012 has reported a hybrid of HKT2;1 and HKT2;2 gene as HKT2;2/1 from Nona Bokra which has strong permeability to Na^+^ and K^+^ even at high external Na^+^ concentrations. Spatial localization and differential expression of these genes further enhances their importance. Horie et al. (2001) reported two isoforms of HKT transporters, a Na + transporter OsHKT1 and a Na^+^- K^+^-coupled transporter OsHKT2, which may act harmoniously in the salt tolerant indica rice. Further studies reported that OsHKT1;4 is expressed around xylem in the leaf sheath while OsHKT1;5 is expressed around the root xylem (Cotsaftis et al. 2011). Similarly, OsHKT2;1 and OsHKT2;4 are expressed in the outer part of the root and in the root hairs and may provide an entry point for Na^+^ into plant roots from the soil (Lan et al. 2010; Schachtman and Schroeder 1994). HKT2;1 expression is significantly upregulated in the root cortex under K^+^ starvation and high Na^+^ concentration (Almeida et al. 2013; Horie et al. 2001). Further, it was proven that the Na^+^ influx into K^+^-starved rice roots was primarily OsHKT2;1-dependent while at concentration >30 mM NaCl, it was permeable to Na^+^ only (Jabnoune et al. 2009). Another class 2 transporter gene *OsHKT2*;*4* shows 93 % similarity with *OsHKT2*;*3* and mediates K^+^ transport independent of Na^+^ concentration (Horie et al. 2011).

The level of salt tolerance provided by known transporter genes in the modern rice cultivars is insufficient and hence new genes or novel allelic variants of these transporters are needed for enhanced salinity tolerance. Crop domestication has selected only a few agronomically desirable alleles and left behind vast pool of genetic diversity in the wild progenitor species due to domestication bottleneck (Tanksley and McCouch 1997). Potential implication of germplasm and crop wild relatives under extreme environment conditions has been reviewed (Mickelbart et al. 2015). Allelic variant of members of HKT transporters such TmHKT1;5-A (Munns et al. 2012), TaKHT1;5-D (Yang et al. 2014) has been introgressed from wild relatives that led to increase in yield of the plant. Allele mining identifies superior alleles from related genotypes that may have been the effect of mutations in the process of evolution. The superior alleles can be used to develop allele specific markers and use them in marker assisted selection and also in tracing the evolution of alleles. Sequencing based allele mining and association analysis is an effective strategy to unravel the hidden potential of wild rice germplasm. Allele mining has been used across the crop species and novel alleles have been identified for abiotic stress tolerance genes in rice (Latha et al. 2004; Negrão et al. 2013; Platten et al. 2013; Singh et al. 2015a), maize (Yu et al. 2010) and barley (Cseri et al. 2011). However, a collection of untapped germplasm is required to mine novel desirable alleles and identify nucleotide sequence variations associated with these alleles (Kumar et al. 2010). India has a wealth of untapped wild rice germplasm that requires hasty expeditions to collect and exploit this fast depleting genetic resource (Singh et al. 2013). The genes already exploited from Indian wild rice include grassy stunt virus resistance from *Oryza nivara* (Khush and Ling 1974), *Bph 19(t)* from *Oryza rufipogon* (Li et al. 2010), *Xa38* from *Oryza nivara* (Bhasin et al. 2012), salinity tolerance genes, inositol methyl transferase (Sengupta et al. 2008) and L-myo-inositol 1-phosphate synthase from wild rice *Porteresia coarctata* (Das-Chatterjee et al. 2006). The male sterility (MS) gene from *O. rufipogon* was introduced into the cultivated rice, leading to development of high yielding hybrid rice (Yuan et al. 1993). The beneficial alleles derived from wild relatives of rice have been transferred into elite genetic backgrounds leading to enhanced trait performance in rice (McCouch et al. 2007; Xiao et al. 1996; Xiao et al. 1998).

In the present study, 299 accessions of rice comprising *O. rufipogon* and *O. nivara* were screened for their level of salt tolerance. A candidate gene based allele mining was used to find natural allelic variants of agronomically important HKT family genes. Genes were re-sequenced with their promoters from 103 accessions of wild and cultivated rice varieties and their nucleotide and haplotype diversity was analysed. SNP based association analysis was done to link traits for salt tolerance e.g., SPAD for chlorophyll, Na^+^ and K^+^ concentration in the shoot and root of rice. Further, merit of *HKT1*;*5* and *HKT2*;*3* were discussed for their potentiality in salt tolerance of rice.

## Results

### Phenotypic Screening of the Wild Rice Germplasm for Salt Tolerance

**Fig. 1:**
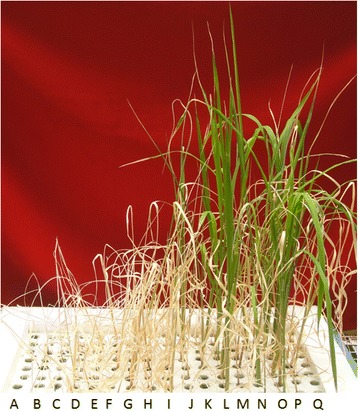
Response of wild rice accessions after 15 days of 150 mM of salt stress in hydroponics. **a** Border; **b** NKSWR149; **c** NKSWR112; **d** NKSWR104; **e** NKSWR092; **f** NKSWR119; **g** NKSWR085; **h** NKSWR115; **i** NKSWR132; **j** NKSWR097; **k** Oryza nivara330646; **l** NKSWR143; **m** NKSWR101; **n** NKSWR079; **o** FL478; **p** VSR156; **q** Border (NKSWR143 is tolerant other accessions are sensitive, FL478- tolerant check, VSR156- sensitive check)

Based on standard evaluation system (SES) of IRRI (Gregorio et al. 1997), 299 wild rice accessions were screened and classified according to their level of salt tolerance (Fig. 1, Additional file 1: Table S1). Two accessions NKSWR173 and NKSWR202 were found highly tolerant with SES score 1 as compared to the tolerant check FL478 with SES score 3 after 15 days of salt stress, 30 accessions were found tolerant at 10 days of salt stress, of which 14 accessions maintained the tolerance comparable to FL478 till fifteenth day. Total 84 accessions were found moderately tolerant, 115 sensitive and 67 highly sensitive at 10 days of salt treatment. However, at fifteenth day only 28 accessions were moderately tolerant, while 69 accessions became sensitive and 186 highly sensitive. One accession, *O. nivara* 336676 displayed interesting result showing high tolerance up to tenth day and thereafter it became highly sensitive and died within the next 2 days in all three years of the experiment (Additional file 1: Table S1). After this primary screening, 45 selected accessions, including all tolerant lines and representative lines from the other sensitive classes, were further evaluated for salt tolerance parameters such as biomass, chlorophyll content (using SPAD meter) and Na + and K+ concentrations in replicated tests. The Na^+^/K^+^ ratio in shoot under stress ranged from 0.8 to 2.1 with the maximum value in *O. rufipogon* 336679 and the minimum in CSR27. The lowest Na^+^/K^+^ ratio (1.3) in the root was observed in a tolerant line NKSWR232 and the highest ratio (3.3) was in NKSWR144. FL478 had highest chlorophyll content while VSR156 and NKS074 had no chlorophyll as the plants died (Additional file 2: Table S2).

### Nucleotide Diversity, Haplotype Diversity and Haplotype Networks of the *HKT* Genes

Eight HKT transporter family genes along with their promoters were re-sequenced from 103 accessions of wild rice and cultivated rice varieties and their nucleotide and haplotype diversity was analysed (Table 1). Nucleotide diversity (π) was the highest (0.00169) for *HKT2;3* and the lowest (0.00012) for *HKT1;1* gene. Maximum haplotype diversity (HD) of 0.891 was found in *HKT2;3* gene followed by *HKT1;4* (0.692) and the lowest in *HKT1;1* (0.084) gene (Table 1). Haplotype analysis showed the maximum 23 haplotypes each for the *HKT1;5* and *HKT2;3* genes with 45 and 28 SNP sites, respectively. The *HKT2;4* gene showed the least number of 5 haplotypes. The Tajima’s D test, Fu and Li’s D* test and Fu and Li’s F* statistics were performed to distinguish between random versus non-random mutations. All the test values were analysed for entire gene, as well as separately for the coding and non-coding regions. Except for the coding region of all accessions, which had positive values of Tajima’s D test, all other regions of the genes among each class individually and in group deviated significantly from neutrality and Tajima’s D test values were negative.

**Table 1:** Nucleotide and haplotype diversity and tests of neutrality for eight high affinity potassium transporter (HKT) family genes from 95 Indian wild rice accessions and 8 cultivated varieties

Candidate gene (Locus ID)	Region	S	π	θω	H	HD	D	D*	F*
HKT1;1	Coding	8	0.00013	0.00084	10	0.217	−2.10133	−3.97252	−3.94393
(LOC_Os04g51820)	Non-coding	3	0.0001	0.00059	4	0.076	−1.48732	−2.01292	−2.16727
	All	11	0.00012	0.00076	13	0.084	−2.22874	−4.21517	−4.17222
HKT1;2	Coding	0	0	0	1	0	0	0	0
	Non-coding	20	0.00027	0.00135	12	0.387	−2.30631	−4.55086	−4.42942
Chr4:30548314..30545885	All	20	0.00026	0.00131	12	0.387	−2.30631	−4.55086	−4.42942
HKT1;3	Coding	8	0.00019	0.00108	9	0.25	−1.99519	−2.80081	−2.99198
(LOC_Os02g07830)	Non-coding	6	0.00067	0.00137	4	0.164	−1.14077	0.15513	−0.32717
	All	14	0.00037	0.00118	10	0.29	−1.88141	−1.8801	−2.24114
HKT1;4	Coding	2	0.00031	0.00091	3	0.128	−1.01249	−1.0804	−1.23948
(LOC_Os04g51830)	Non-coding	42	0.00059	0.00191	19	0.69	−2.1536	−3.07965	−3.2462
	All	44	0.00057	0.00182	20	0.692	−2.15775	−3.12193	−3.27713
HKT1;5	Coding	8	0.00025	0.00183	8	0.166	−2.08122	−3.61227	−3.65909
(LOC_Os01g20160)	Non-coding	37	0.0012	0.00322	18	0.571	−1.94371	−2.70539	−2.87895
	All	45	0.00094	0.00284	23	0.631	−2.10258	−3.40294	−3.44926
HKT2;1	Coding	5	0.00015	0.00073	5	0.18	−1.66589	−3.23816	−3.20982
(LOC_Os06g48810)	Non-coding	46	0.00042	0.00241	17	0.333	−2.60346	−4.60994	−4.55342
	All	51	0.00035	0.00196	18	0.421	−2.61003	−4.91693	−4.76765
HKT2;3	Coding	24	0.00238	0.00353	23	0.891	−0.96284	−1.72038	−1.71118
(LOC_Os01g34850)	Non-coding	4	0.00013	0.00132	3	0.038	−1.77201	−3.90552	−3.78567
	All	28	0.00169	0.00284	23	0.891	−1.22676	−2.67757	−2.5308
HKT2;4	Coding	0	0	0	1	0	0	0	0
(LOC_Os06g48800)	Non-coding	4	0.0012	0.00448	5	0.198	−1.44156	−1.48409	−1.72999
	All	4	0.0012	0.00448	5	0.198	−1.44156	−1.48409	−1.72999

(*S* SNP/Indel sites, π nucleotide diversity, θω nucleotide diversity with Watterson’s estimator, *H* number of haplotypes, *HD* haplotype diversity, *D* Tajima’s D statistic, D* Fu and Li’s D* statistic, F*: Fu and Li’s F* statistic.)

**Fig. 2:**
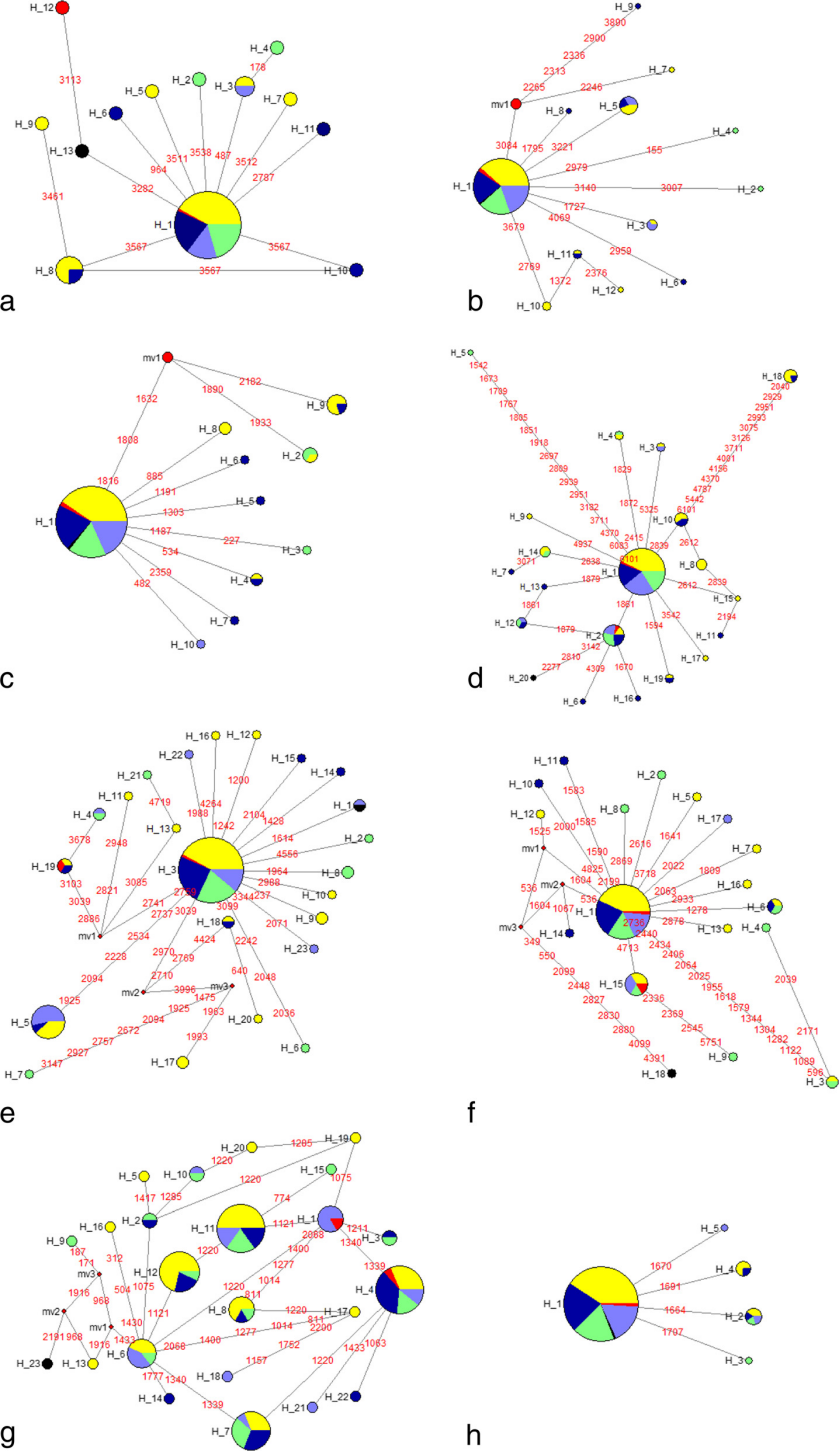
Haplotype networks of eight HKT transporter genes including (**a**) *HKT1;1* (**b**) *HKT1;2* (**c**) *HKT1;3* (**d**) *HKT1;4* (**e**) *HKT1;5* (**f**) *HKT2;1* (**g**) *HKT2;3* (**h**) *HKT2;4*. Each circle represents a haplotype and size of each circle is proportional to haplotype (allele) frequency. Colour coding represents phenotype class based on standard evaluation system (SES) score. (Red-highly tolerant, Purple-tolerant, Green moderately tolerant, Blue-Sensitive, Yellow-highly sensitive)

Haplotype analysis of *HKT1;1* gene showed 13 haplotypes with haplotype number H12 having a single highly tolerant accession and a distinguishing guanine residue instead of adenine in the first intron. *HKT1;2* gene has diversified to 13 haplotypes, *HKT1;3* into 10 haplotypes and *HKT1;4* to 20 haplotypes. Network analysis of *HKT1;5* gene showed that out of the total 23 haplotypes of this gene two haplotypes, H5 and H22 included all the tolerant accessions. Haplotype H5 had 13 accessions, including tolerant cultivated varieties FL478 and CSR27 along with sensitive variety VSR156 and five sensitive accessions from Gujrat. A unique haplotype H22 was represented by salt tolerant cultivated rice variety CSR11, which also matched with the published sequence of tolerant Indian rice variety Nona Bokra (Ren et al. 2005). The members of HKT sub-family 2 genes, *HKT2;1* and *HKT2;4* showed 18 and 5 haplotypes, respectively. Further, haplotype analysis of *HKT2;3* gene showed that its haplotype H1 included four tolerant wild rice accessions along with the salt tolerant check varieties FL478 and CSR27. However, another salt tolerant variety CSR11 possessing haplotype H10 grouped separately along with tolerant *O. nivara* accession 330641. A phenotypically unique accession, showing tolerance only up to 10 days of stress followed by extreme sensitivity, had a unique haplotype H5 of the *HKT2;3* gene (Fig. 2).

### Evolution of the HKT Family Genes

**Fig. 3:**
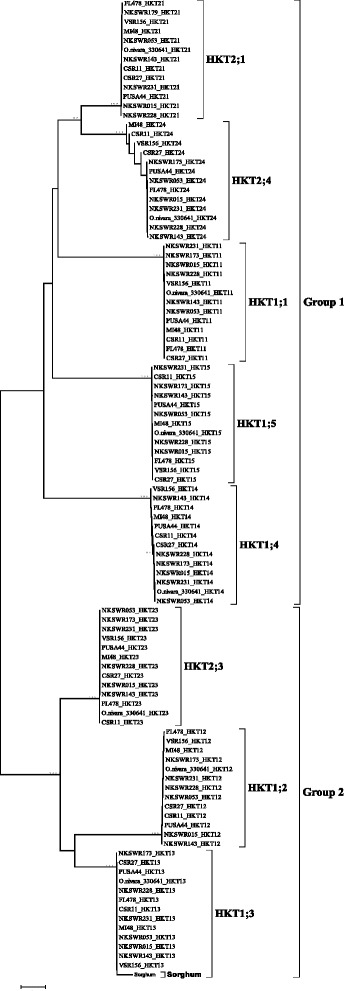
Minimum evolution phylogenetic tree of eight *HKT* genes re-sequenced from representative wild rice accessions and cultivar checks constructed using MEGA 5.1 software

A phylogenetic tree was constructed using nucleotide sequences of the eight HKT family genes using NJ method. Sorghum *HKT* gene taken from sorghum gene database was included as outgroup. As expected the tree showed total eight clades representing the individual HKT genes that clustered into two major groups. The phylogenetic tree revealed a somewhat different relationship among the HKT transporter genes as compared with their functional classification. The *HKT1;3* gene is at the root of divergence as it was most closely related to the sorghum *HKT* gene. A close relationship was observed between the *HKT1;3, HKT1;2* and *HKT2;3.* The remaining five genes were clustered in group I and showed that *HKT1;4* was most primitive followed by *HKT1;5* and *HKT1;2.* The recently evolved genes *HKT2;4* and *HKT2;1* are located in tandem on chromosome 6 and very closely related to each other diverging from a common branching point (Fig. 3).

### Association of HKT Gene SNPs with Salt Tolerance

**Fig. 4:**
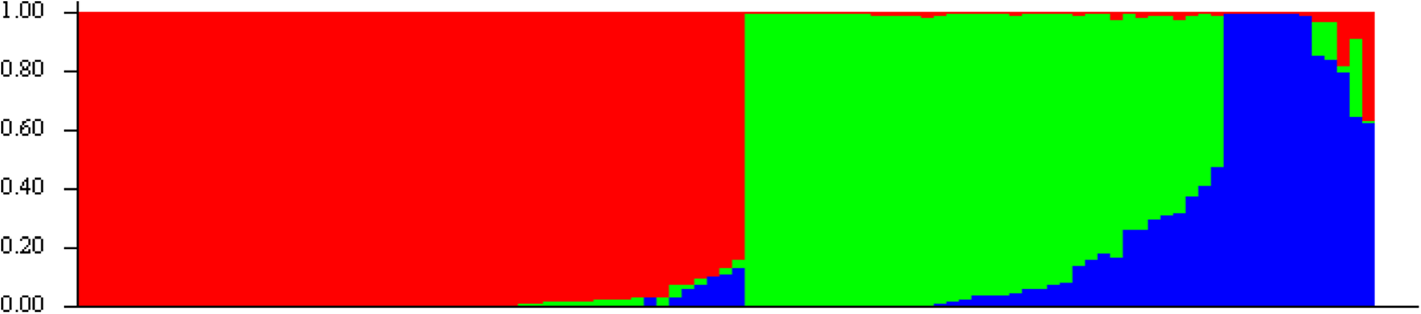
Distribution of 103 wild rice accessions and cultivated rice into three different sub-populations based on 48 genome wide SNP markers. X-axis shows rice accessions and y-axis represents Fst values (*Blue*: Population I; *Green*: Population II; *Red*: Population III)

The association study was conducted on 45 accessions including 3 check rice varieties, of which, 18 belonged to tolerant class, 9 moderately tolerant, 7 sensitive and 11 highly sensitive. For association study a Bayesian based analysis of population structure was conducted which used an ad hoc statistics (∆K), the rate of change in log probability of data between successive K values. It showed that the highest log likelihood was at *K* = 3 suggesting three major sub-population in the analysed Indian wild rice accessions. The individual assignments and Q-matrix in the three different sub-population revealed that sub-population I included 51.4 %, sub-population II 33.8 % and sub-population III 14.8 % of the analysed accessions. The mean Fst values were 0.8981, 0.6154 and 0.7291 for the sub-populations I, II and III, respectively (Fig. 4, Additional file 3: Table S3).

SNP based association analysis showed significant associations with the salt tolerance traits using MLM_Q + K statistical method. Thus, the results were filtered with association values greater than 5 % and p value less than 0.01. A total of 50 SNPs from all the eight *HKT* genes showed significant associations with the salt tolerance traits (Table 2, Additional file 4: Figures S1, S2).

**Table 2:** Association of SNPs with different salt related traits at 10 days of treatment with HKT genes (with R2 > 5 % and *p* < 0.01)

Gene	Trait	SNP	Site	*p*	R2 (%)	SNP type	MAF	Gene	Trait	SNP	Site	*p*	R2 (%)	SNP type	MAF
HKT 1;1	N_R	S0231	30724332	0.003	34.43	Int	37	HKT 1;5	N_S	S0077	11459042	0.002	31.57	Int	36
	K_S	S1955	30726056	0.003	30.75	Syn	5		N_S	S3893	11462858	0.005	33.00	P/A_140_	20
	SPAD	S2094	30726195	0.003	31.90	S/N_258_	32		K_S	S0077	11459042	0.003	30.92	Int	36
	SPAD	S2331	30726432	0.003	31.90	S/F_179_	30		N_R	S0887	11459852	0.003	34.12	Int	13
	SPAD	S2546	30726647	0.003	31.90	Syn	30		N_R	S0919	11459884	0.009	34.42	Int	35
HKT 1;2	K_S	S0221	30545988	0.003	31.83	5’ NC	36		N_R	S0958	11459923	0.009	34.65	Int	35
	K_S	S1430	30547197	0.009	25.16	Int	21		N_R	S0959	11459924	0.003	34.12	Int	13
	K_S	S2282	30548049	0.003	31.54	Int	31		N_R	S0974	11459939	0.008	35.09	Int	6
HKT 1;3	N_R	S0337	4103487	0.005	33.93	C/R_16_	30		N_R	S1036	11460001	0.002	37.37	Int	5
	SPAD	S0091	4103241	0.009	27.83	5’ NC	12		K_R	S1036	11460001	0.008	26.80	Int	5
	N_R	S0744	4103894	0.008	38.19	Syn	22		N_R	S1379	11460344	0.003	34.44	K/Q_429_	49
	SPAD	S0744	4103894	0.008	35.21	Syn	22		N_R	S1913	11460878	0.003	34.62	Int	43
	SPAD	S1682	4104832	0.009	27.83	Int	10		N_R	S2043	11461008	0.004	33.62	Int	29
	SPAD	S1762	4104912	0.005	31.75	Int	45		K_S	S2146	11461111	0.002	34.93	Int	45
	SPAD	S2347	4105497	0.003	34.78	A/V_488_	23		K_S	S2347	11461312	0.006	27.43	Int	28
	SPAD	S2374	4105524	0.004	32.49	R/Q_497_	46		K_S	S2865	11461830	0.001	36.63	Int	14
	SPAD	S2392	4105542	0.009	27.68	T/M_503_	13		K_S	S3893	11462858	0.009	31.17	Int	20
HKT 1;4	SPAD	S0886	30734275	0.003	34.72	3’NC	32	HKT 2;1	K_S	S3062	29541671	0.008	32.35	5’ NC	7
	SPAD	S1267	30734656	0.003	34.72	3’NC	32		N_R	S3062	29541671	0.004	39.70	5’ NC	7
	SPAD	S2284	30735673	0.002	37.42	Int	32		SPAD	S4153	29542762	0.009	25.80	5’ NC	38
	SPAD	S2804	30736193	0.003	34.72	Int	32		SPAD	S4377	29542986	0.002	33.96	5’ NC	36
	SPAD	S2974	30736363	0.002	37.03	Int	33		K_S	S5815	29544424	0.002	40.52	5’ NC	21
	SPAD	S3185	30736574	0.002	37.03	Int	32		K_S	S5817	29544426	0.007	32.66	5’ NC	7
	SPAD	S3228	30736617	0.007	36.36	Int	33	HKT 2;3	K_S	S0356	19241884	0.002	34.84	Int	10
	SPAD	S3307	30736696	0.003	34.87	Int	28		SPAD	S0761	19242289	0.003	31.88	I/T_77_	34
	SPAD	S3544	30736933	0.003	34.72	Int	32		K_S	S1001	19242529	0.009	25.08	I/T_157_	5
	SPAD	S4678	30738067	0.003	35.09	Int	35		SPAD	S2053	19243581	0.003	31.79	Syn	34
								HKT 2;4	K_S	S0334	29534433	0.004	37.20	Int	37

(*N_S* sodium concentration in shoot, *K_S* potassium concentration in shoot, *N_R* sodium concentration in root, *K_S* potassium concentration in root, *Int* intron, *Syn* synonymous, *NC* non-coding, *MAF* minor allele frequency in %)

Among the HKT subfamily I members, analysis of associated SNPs of *OsHKT1;1* gene revealed two non-synonymous substitutions, (i) S2094 (Ser_258_ to Asp_258_) and (ii) S2331 (Ser_179_ to Phe_179_) that were significantly associated with SPAD chlorophyll content. These two SNPs are spatially located, one (S2094) on the extracellular side and the other on the cytoplasmic side (S2331). In addition, one SNP (S0231) in the intronic region showed highly significant association with sodium concentration in the root. Further, two synonymous SNPs were associated with potassium concentration in the shoot (S1955) and SPAD chlorophyll content (S2546). In case of *HKT1;2* gene three SNPs were associated with potassium concentration in the shoot, two of these (S1430 and S2282) were present in the intronic region while third one (S221) was in the upstream promoter region. In case of *OsHKT1;3* a non-synonymous SNP S337 (Cys_16_ to Arg_16_) present in the extracellular domain of the protein explained 34 % of phenotypic variance (R^2^ value) and was associated with sodium concentration in the root while, three non-synonymous SNPs in the cytoplasmic domain, S2374 (Ala_488_ to Val_488_), S2374 (Arg_479_ to Glu_479_) and S2392 (Thr_503_ to Met_503_) showed association with SPAD chlorophyll content. A synonymous SNP (S744) was associated with potassium concentration in the shoot and SPAD chlorophyll content. Interestingly, the SNP in the promoter region (S91) and those in the intronic region (S1682 and S1762) were also associated with the SPAD chlorophyll value. All SNPs identified in the *OsHKT1;4* gene were associated with SPAD chlorophyll content, among them, two were in the downstream promoter region while eight were in the intronic region. Of the two SNPs in the downstream promoter region (S886 and S1267), one was in the consensus GT-1 binding site and the other one in the ACGT sequence. In the *OsHKT1;5* gene total 14 SNPs were associated with the salt tolerance traits. Of which 12 were in the intronic region while remaining 2, S1379 (Lys_429_ Glu_429_) and S3893 (Pro_140_ Ala_140_), were non-synonymous. Both the non-synonymous SNPs were associated with sodium concentration: S1379 was located on extracellular side and showed significant association with the concentration of sodium in the root while, the other S3893 was on the cytoplasmic side and showed association with concentration of sodium in shoot, explaining 32 % (R^2^ value) of the phenotypic variance (Table 2).

From the HKT subfamily II, *OsHKT2;1* gene had five SNPs in the promoter region showing association with the salt tolerance traits. Three of these (S3062, S5815, and S5817) were associated with shoot potassium concentration, of which S3062 was also associated with root sodium concentration, while the other two, S4153, and S4377, were associated with SPAD chlorophyll content. In *OsHKT2;3* a total of 4 SNPs were associated with the salt tolerance traits. Of the two non-synonymous SNPs, S1001 was associated with potassium concentration in shoot (extracellular side) while S761 was associated with SPAD chlorophyll content (cytoplasmic side), both the SNPs resulted in Isoleucine to Threonine substitution at amino acid positions 157 and 77, respectively. In addition, one synonymous SNP S2053 was associated with SPAD chlorophyll content and another SNP S356 in the intronic region was associated with potassium concentration in shoot. Only one SNP S334 present in the intronic region of *OsHKT2;4* gene was associated with potassium concentration in the shoot (Table 2).

## Discussion

Wild rice is expected to play important role in rice improvement in the coming years. In this study we explored untapped diversity of Indian wild rice to identify natural alleles of the HKT transporter family genes. Analysis of nucleotide sequence variations for eight HKT family genes in wild rice showed higher nucleotide and haplotypic diversity as compared to the cultivated rice varieties (Table 1, Fig. 2). This supports the notion that wild relatives are genetically much more diverse than their cultivated counterparts (Hoisington et al. 1999). However, nucleotide variations of a gene is also associated with respect to position in the gene (McNally et al. 2006). Platten et al. (2013) observed comparatively lower nucleotide diversity and haplotypic diversity in cultivated rice and identified relationship between different haplotypes and salt tolerance for *HKT1;5* gene. Our results indicate that nucleotide diversity was quite different from haplotype diversity as only effective SNPs participated in haplotypic group determination (Goodall-Copestake et al. 2012). Differences between nucleotide and haplotype diversity has been measured across different genes such as Sucrose Synthase 3 in rice (Lestari et al. 2011) and *OsDREB1F* in wild and cultivated rice (Singh et al. 2015a).

To understand the natural selection process in the evolutions of HKT genes, Tajima’s D test, Fu and Li’s D* test and Fu and Li’s F* statistics were calculated. Negative values obtained for wild rice population shows an excess of rare polymorphisms which still undergoes some population expansion and positive selection pressure and were further validated by Ka/Ks ratio and Jukes and Counter correction values (>1). However, positive value for coding region in cultivated rice revealed that alleles had evolved either by balancing selection or population bottlenecks (Table 1, Akey et al. 2004).

Among the eight genes only two *HKT1;5* and *HKT2;3* showed tolerant haplotypes. Functionally, HKT1;5 is a Na^+^ specific transporter that maintains Na^+^/K^+^ homeostasis in the leaves under salt stress (Ren et al. 2005; Mickelbart et al. 2015), whereas HKT2;3 regulates K^+^ transport independent of Na^+^ concentration (Horie et al. 2011). The genotypes having tolerant allele of *HKT1;5* showed lower Na^+^/K^+^ concentration in the shoot as compared to root (Thomson et al. 2010). Six wild rice accessions that showed lower shoot Na^+^ grouped into four separate haplotypic group. In addition, four other accessions that showed low Na^+^ concentration in shoot comparable to the tolerant checks fell into separate haplotypic groups indicating allelic variation for the *HKT1;5* gene among the accessions (Fig. 2, Additional file 2: Table S2). An exceptionally higher Na^+^ and K^+^ ratio was observed in the shoots of a tolerant accession *O. rufipogon* 336679 indicating presence of an alternate mechanism to sustain such a high level of salt concentration in shoot. Mian et al. (2011) identified role of *HKT 2;1* gene in barley and reported increased shoot Na + concentrations and improved biomass production under salt stress. Haplotype analysis of *HKT2;3* gene showed almost even distribution of accessions into different haplotypic groups, but it also formed a separate haplotype for the reference sequence. Of the total 23 observed haplotypes, one was associated with tolerant and two were associated with moderately tolerant genotypes. A unique accession of *O. nivara 336676* showing tolerance only up to tenth day of applying the stress separated in a different haplotype indicating that it is possibly following some different tolerance mechanism which breaks down beyond 10 days of stress. Salt susceptibility of this accessions after 10 days might be due to higher accumulation of Na^+^ in root (Additional file 2: Table S2). Another plausible explanation for the tolerance in this accession is presence of root specific tonoplast transporters with some accessory factors associated with the genes for providing energy to transport ions into vacuole but with limited accumulation capacity of ions in vacuole. Functionally, HKT2;3 mediates K^+^ transport independent of Na^+^ concentration hence, even at high salt concentration the physiological functions that are vital to crop plants relating to K^+^ e.g., stomatal movement, CO_2_ uptake and cofactor to make enzymes etc. are maintained inside the cell. It also maintains ionic homeostasis (Horie et al. 2007; Roy et al. 2014). However, haplotype analysis revealed uneven distribution of tolerant genotypes among different haplotypic group for different genes. It shows that an unknown type of mechanism might be present among the Indian wild rice that could help to enhance our understanding towards salt tolerance mechanism and be used for improvement of cultivated rice.

Phylogenetic analysis describes the evolutionary relationship among genes and different clustering patterns indicate the level of their functional divergence (Mäser et al. 2001). Here, two genes of phylogenetic group I (*HKT2;1* and *HKT 2;4*) showed high nucleotide similarity with each other. They most likely have evolved from recent duplication of a single gene as also suggested by their tandem location in the rice chromosome 6. Interestingly, in the phylogenetic tree functionally diverse, *HKT1;3* and *HKT1;2* genes grouped together along with *HKT2;3*. This suggests that members of functionally different groups might have evolved from a common ancestor through duplication and subsequent functional divergence.

Population structure analysis revealed three distinct subpopulations in the Indian wild rice accessions with extensive genetic variation between and also within the populations as was also shown recently using a high density 50 K SNP chip (Singh et al. 2015b). Higher average values of Fst indicated that a high level of unshared allelic variation was present in the population (Holsinger and Weir 2009). Here sub-population I comprising more than 50 % of the wild rice accessions was the largest of the three sub-populations.. Linkage disequilibrium (LD) based association analysis is supposed to be the best method for association analysis (Yu and Buckler 2006). Here, LD based association analysis showed strong associations of multiple SNPs in different candidate *HKT* genes with the salt tolerance traits.

Haplotype based association analysis for identification of allelic variants associated with specific traits is considered better over single-allele studies (Johnson et al. 2001). Overall, only three tolerant haplotypes were identified, one for the *HKT1;5* gene and two for the *HKT 2;3* gene, but, multiple SNPs were found to be associated with different salt tolerance traits. It may be because of less number of accessions grouping in many haplotypes resulting in low minor allele frequencies (<2 %), and synonymous or noncoding nature of the SNPs. Negrão et al. (2013) identified allelic variants for *HKT1;5* gene by setting filter for associated SNPs and individually assessing the results. Here total 14 filtered SNPs were found to be associated with traits for *HKT1;5*, and among them 12 SNPs were in the intronic regions, one was synonymous and two were non- synonymous, S1379 (Lys_429_ to Glu_429_) and S3893 (Pro_140_ to Ala_140_) leading to amino acid substitutions. Replacement of proline causes changes in the protein backbone that is responsible to introduce tight turns or kinks into alpha helices (Betts and Russell 2003). Conversion of proline to alanine was also found in Pokkali and Nona Bokra alleles of the *HKT1;5* gene which is supposed to increase the substrate specificity (Cotsaftis et al. 2012; Negrão et al. 2013). Cotsaftis et al. (2012) reported change in conformation of protein with change in overall charge.

Two SNPs in the promoter region of *OsHKT1;4* gene, S886 (GT-1 binding site region) and S1267 (downstream to the promoter ACGT sequence), were significantly associated with SPAD chlorophyll content value. It has been reported that in many light-regulated genes like *PHYA* in oat and rice are regulated by GT-1 binding site (Villain et al. 1996). Further, Park et al. (2004) has reported this site to be salt induced. Downstream to the promoter region is the ACGT sequence that is required for etiolation induced expression of *erd1* (early responsive to dehydration) gene in *Arabidopsis* (Simpson et al. 2003), hence it is speculated that *OsHKT1;4* gene may regulate dehydration stress imposed by salinity.

Two haplotypes (H1 and H10) of the *OsHKT2;3* were associated with salt tolerance SES score. In addition, total 4 SNPs were associated with the analysed salt tolerance traits, one synonymous, one intronic and two non-synonymous SNPs, S761 and S1001, both leading to Iso to Thr substitution at amino acid positions 77 and 157, respectively (Table 2). The isoleucine to threonine substitution is known to impact post translation modifications such as phosphorylation. An increase in the phosphorylation level has been observed with increase in Thr in a protein sequence (Vlad et al. 2008). The non-synonymous mutations outside functional domain of genes may alter structure of the protein and consequently its function (Negrão et al. 2013). Both synonymous and non-synonymous SNPs showed significant association with salt tolerance traits, perhaps affecting the RNA splicing, mRNA stability, and post-translational modification of protein function (Negrão et al. 2013). Some of this could be due to close linkage of a non-functional SNP with the functional SNP. A large number of rare alleles and haplotypes were observed for different HKT genes, whose association with trait could not be studied due to low minor allele frequency. Rare alleles contributing to the gain or enhancement of the trait value may be useful for future adaptability of the rice crop, these may involve novel mechanisms of salt tolerance. Introgression of rare alleles through marker-assisted backcross breeding (MABB) techniques may help develop new genetic resources for breeding of rice for tolerance to extreme salt stress. To find out effective rare alleles, bi-parental mapping populations involving these lines will be needed to validate the function of rare alleles and also to understand their genetic control mechanisms (Semagn et al. 2010).

## Conclusions

The wild rice accessions screened in this study have been collected from different ecological habitats including salt-affected areas. An accessions (NKSWR173) from upland and another accession (NKSWR202) from upper gangetic plain region showed high level of tolerance phenotypic patterns and may have different salt tolerance mechanisms. Haplotype analysis indicated a substantial level of natural diversity for the HKT family of genes, especially for the HKT1;5 and HKT2;3 genes among the Indian wild rice accessions. The novel haplotypes showing association with salt tolerance may have great impact on rice salinity breeding. A programme for introgression of the identified haplotypes into high yielding but salt sensitive rice varieties has been initiated. It will help breed rice genotypes with higher level of salt tolerance beyond the existing salt tolerant varieties.

## Methods

### Plant Material

A total of 299 wild rice accessions including 244 new accessions collected from different geographical regions of India along with their passport data and 58 accessions from NBPGR gene bank, New Delhi, India were analysed. In addition, 6 cultivated *O. sativa* (salt tolerant FL478, CSR27, CSR11 with different tolerance mechanisms, MI48 as a moderate check while VSR156 and Pusa 44 as sensitive checks) were used. The collection sites and other detailed information on each wild rice accession is available in a database at http://nksingh.nationalprof.in/ (Additional file 1: Table S1).

### Phenotyping for Salt Tolerance

Phenotyping for all the accessions was done at National Phytotron Facility, IARI, New Delhi, India for three times. Experiment was planned according to IRRI protocol with minor modifications (Gregorio et al. 1997). Each set of experiment had three replicates with 10 plants of each accession per replicate. The seeds were germinated in petri plates and then transferred into thermocol trays after two days of germination. Each tray had a positive check and a negative check. The seedlings were allowed to grow for 14 days in Hoagland’s solution and on 15th day, they were transferred to Hoagland’s solution with 150 mM NaCl concentrations. Rice seedlings were allowed to grow and scoring was done after 10 days and 15 days of stress according to the 1–9 scale of Standard Evaluation System (SES) developed by Gregorio et al. (1997). Most of the SES were same for all the replicates but in case of variation mode was taken. Based on the above data and geographical location 45 representative accessions from each class were selected to evaluate physiological parameters to reduce the cost and labour. Sodium and Potassium ion concentrations from roots and shoots were estimated using a flame photometer LABTRONICS model LT66 after digestion of 0.1 gm of dried plant sample with 1:3 ratio of perchloric acid and nitric acid. Chlorophyll content of leaves was determined using SPAD meter.

### PCR Amplification and re-Sequencing

After phenotypic screening of 299 accessions, 103 were selected based on their response to salt stress. All the salt tolerant lines and representative accessions from moderately tolerant and sensitive classes were taken for re-sequencing of *HKT* genes. Genomic DNA was extracted from leaf tissue using the CTAB method described by Murray and Thompson (1980). Eight *HKT* genes were amplified using primer walking method (Additional file 5: Table S4). Nucleotide sequence of the genes were retrieved from NCBI database (http://www.ncbi.nlm.nih.gov/) and primers were designed using Primer3 software (http://bioinfo.ut.ee/primer3-0.4.0/). Specific amplification and validation of the primers was done by NCBI Primer BLAST (http://www.ncbi.nlm.nih.gov/tools/primer-blast/) against *Oryza* taxon and high stringency conditions during PCR amplification. The PCR amplifications were carried out in 25 μl reaction consisting of 1 Unit SpeedStar™HS DNA Polymerase from TAKARA BIO INC, 1x Fast Buffer, 2 μl of dNTP mixture, 0.5 Pico moles of each primers and 80 ng of template DNA. The PCR reaction was carried out in BIORAD Thermal cycler under following conditions: Initial denaturation at 98 °C for 3 min followed by 38 cycles of denaturation for 10 s at 98 °C, annealing for1 min at 64 °C and extension for 1 min at 68 °C, and for final extension for 10 min at 68 °C. The amplified products were further checked by electrophoresis in 1 % agarose gel in 1X TBE buffer.

PCR products were directly sequenced by Ion Torrent PGM sequencer (Life Technologies) after fragmentation, library preparation, purification and cycle sequencing according to manufacturer’s instructions. Briefly, the amplicons of different genes of the same genotype were pooled in equimolar concentration, sheared to size of ~200 bp and then barcoded to identify individual accessions. Since each gene has unique sequence no barcoding was needed for individual gene. The sheared and barcoded products were now size selected, pooled together in equi-molar ratio and then PCR sequenced. At each step purification was done using Agencourt AMPure XP reagent using Kingfisher Flex. Ion OneTouch2™ was used for emulsion PCR to clone the library on the beads and thereafter for enrichment. The enriched library was loaded on Ion PGM 316 chip and sequencing was performed on Ion PGM™ sequencer.

### Sequence Data Analysis

The depth of sequencing obtained was approximately 88x. Coverage analysis and variant caller plugins were run and the sequences were viewed in IGV (Robinson et al. 2011; Thorvaldsdóttir et al. 2012). SamTools mpileup command was used to generate consensus sequences against the reference for each gene and alignment of *OsHKT* genes was done by ClustalW (Thompson et al. 1994) in BioEdit (Hall 2011). Sequences were deposited to NCBI GenBank database (KT795544-KT796361). Nucleotide polymorphisms were analysed using the DnaSP software version 5.10 (Rozas et al. 2003). Level of silent-site nucleotide diversities per site (π) (Nei 1987) and population mutation parameter (θ) (Watterson 1975) was estimated. Sliding window analysis was performed to examine nucleotide polymorphism across the genes in all accessions using DnaSP software. Statistical tests of neutrality such as Tajima’s D (Tajima 1989), Fu and Li’s D* and F were calculated to examine the selection pressure at this locus. A haplotype network was constructed for comparison of genealogical relationships among the haplotypes using Network software (Bandelt et al. 1999) (http://www.fluxus-engineering.com). The nucleotide sequences were translated into amino acid sequences, and the protein variants were identified as compared with the reference protein. The bootstrap consensus tree inferred from 500 replicates was constructed to represent the evolutionary history of the HKT genes by using the Minimum Evolution Method in MEGA5 (Tamura et al. 2011).

### Population Structure and SNP-Trait Association Analysis

Association analysis was performed with the MLM model, considering both kinship (K) and population structure (Q), implemented in TASSEL software. The kinship (K) and population structure (Q) were generated from a genome wide 48-plex Illumina GoldenGate SNP genotyping assay (B. Singh, unpublished) and analysing data with Structure software (Pritchard et al. 2000). To overcome the problems of interpreting the real value of K, a range of ad hoc K values were tested and analysed using Evanno plot (Evanno et al. 2005). The results of estimated likelihood values for a given K (from 2 to 10) in five independent runs were harvested with structure harvester an online program (Earl 2012). For association mapping filtered sites within *OsHKT* genes were used to determine linkage disequilibrium (LD) by correlation between alleles at two loci in TASSEL 5.0 (Bradbury et al. 2007) software and significance of LD among SNPs was determined by Fisher’s exact test. The mixed model showed least deviation of observed *P*-values from expected *P*-values in Q-Q plot when compared with that of Q (population structure) or K (kinship) model only. A probability value of 0.01 was used as the threshold for significance of SNP–trait associations. Functions of associated sites in the promoter region was identified by PLACE (Higo et al. 1999).

## Additional files

Additional file 1: Table S1. List of wild rice accessions, their place of collection and Standard Evaluation System (SES) score obtained during screening. (CSV 13 kb) Additional file 2: Table S2. Phenotype (SES) score and evaluated salt related traits among Indian wild rice at 10th day of 150mM salt treatment. (CSV 3 kb) Additional file 3: Table S3. Set of overlapping primers used for amplification of HKT genes. (CSV 3 kb) Additional file 4: Figure S1. Linkage Disequilibrium plots for HKT genes **Figure S2.** Q-Q plots of HKT genes obtained after MLM based association of SNP with traits. (PDF 1156 kb) Additional file 5: Table S4. List of varieties and their classification in different sub-populations using Structure software. (CSV 1 kb)
