# Supplementary material for: Association of SNP Haplotypes of HKT Family Genes with Salt Tolerance in Indian Wild Rice Germplasm
Source: Rice (N Y). 2016 Mar 29;9:15. doi: 10.1186/s12284-016-0083-8 (PMC4811800; doi:10.1186/s12284-016-0083-8)
Supplement: Additional file 4: Figure S1. — Linkage Disequilibrium plots for HKT genes Figure S2. Q-Q plots of HKT genes obtained after MLM based association of SNP with traits. (PDF 1156 kb) [file 12284_2016_83_MOESM4_ESM.pdf]

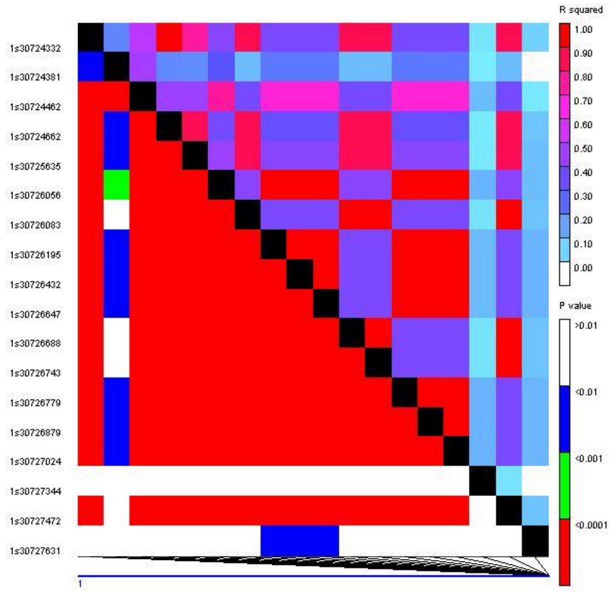

a) HKT1;1

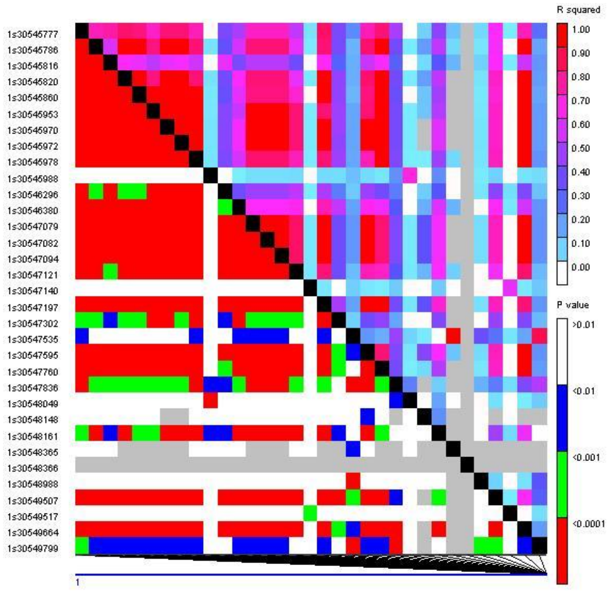

b) HKT1;2

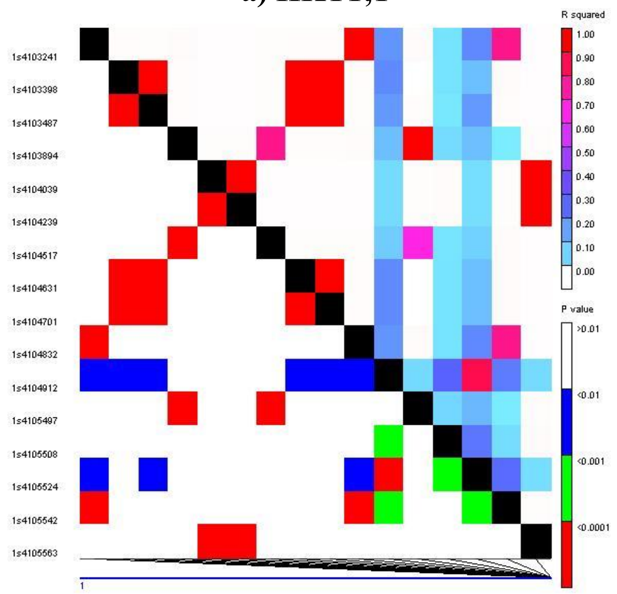

c) HKT1;3

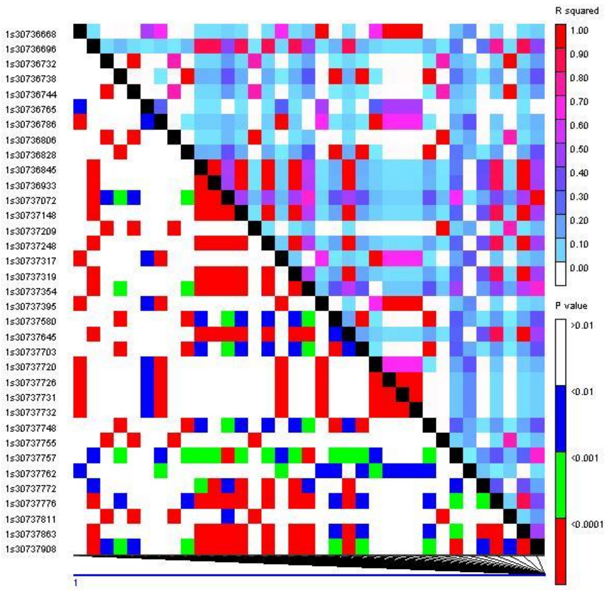

d) HKT1;4

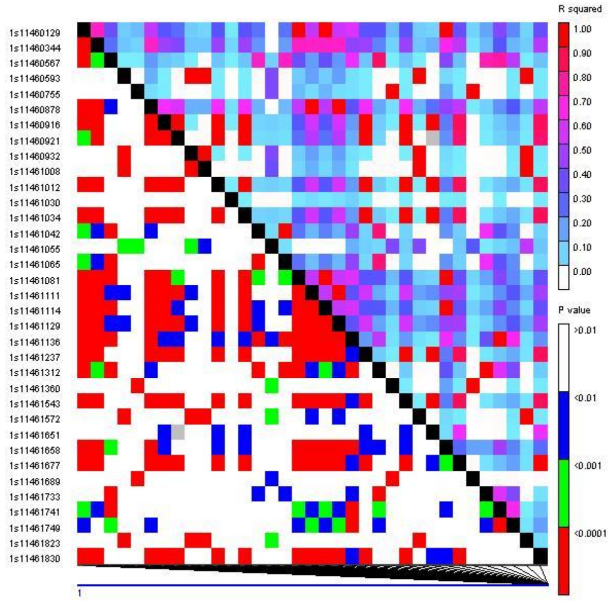

e) HKT1;5

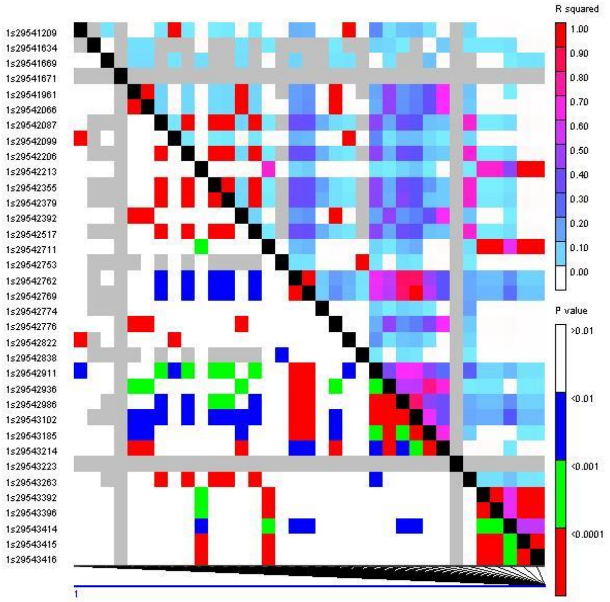

f) HKT2;1

(Continued from page 1)

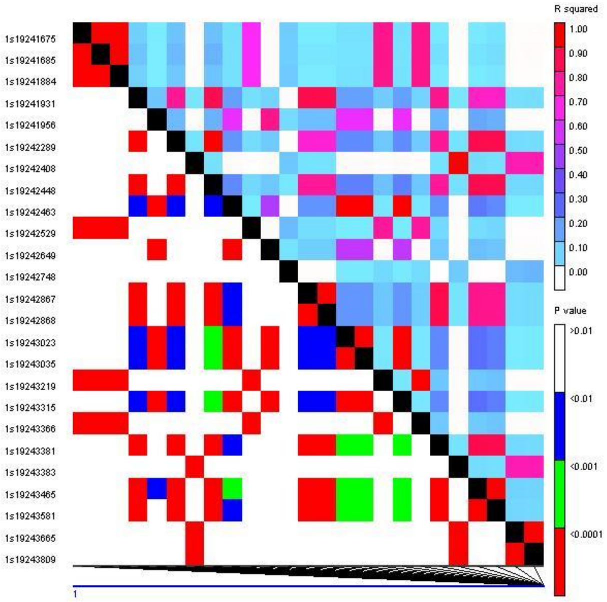

g) HKT2;3

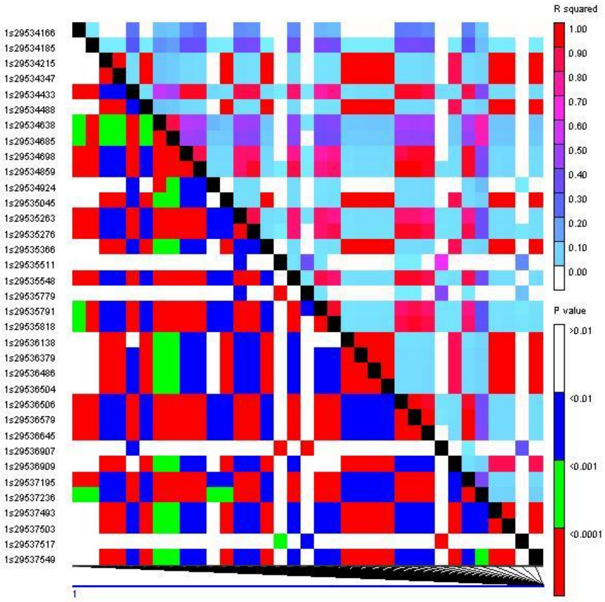

h) HKT2;4

Supplementary Figure 1: Linkage Disequilibrium plots for HKT genes

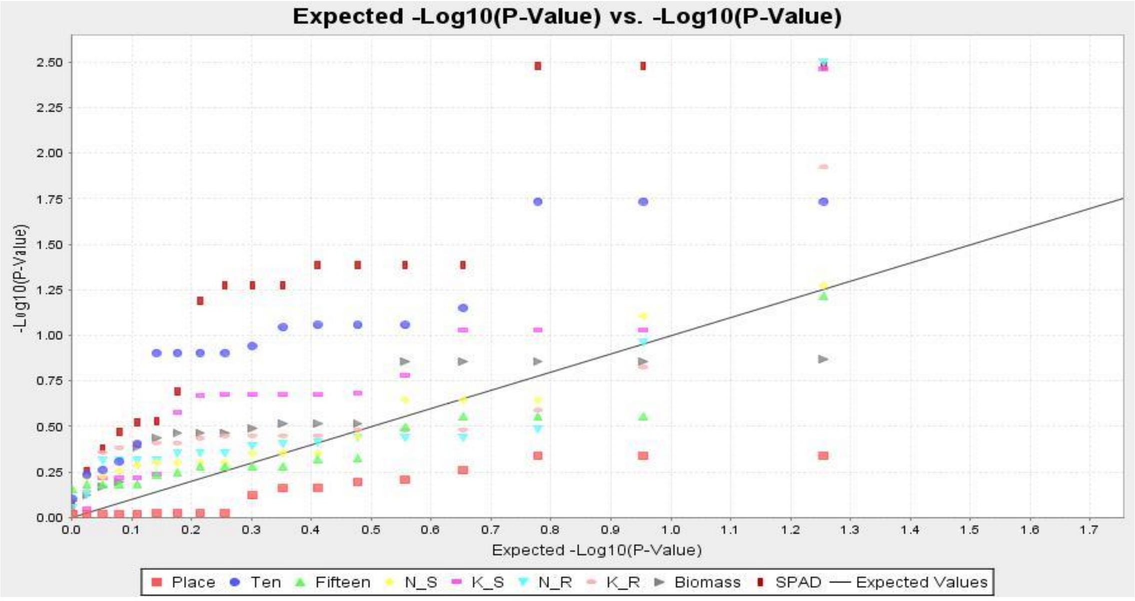

a) HKT1;1

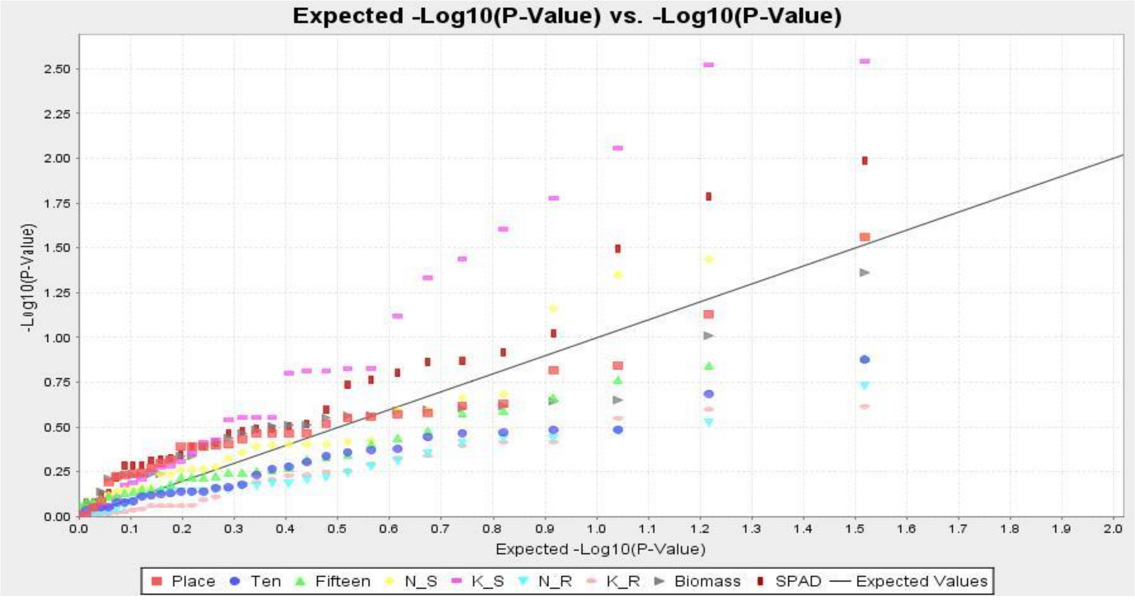

b) HKT1;2

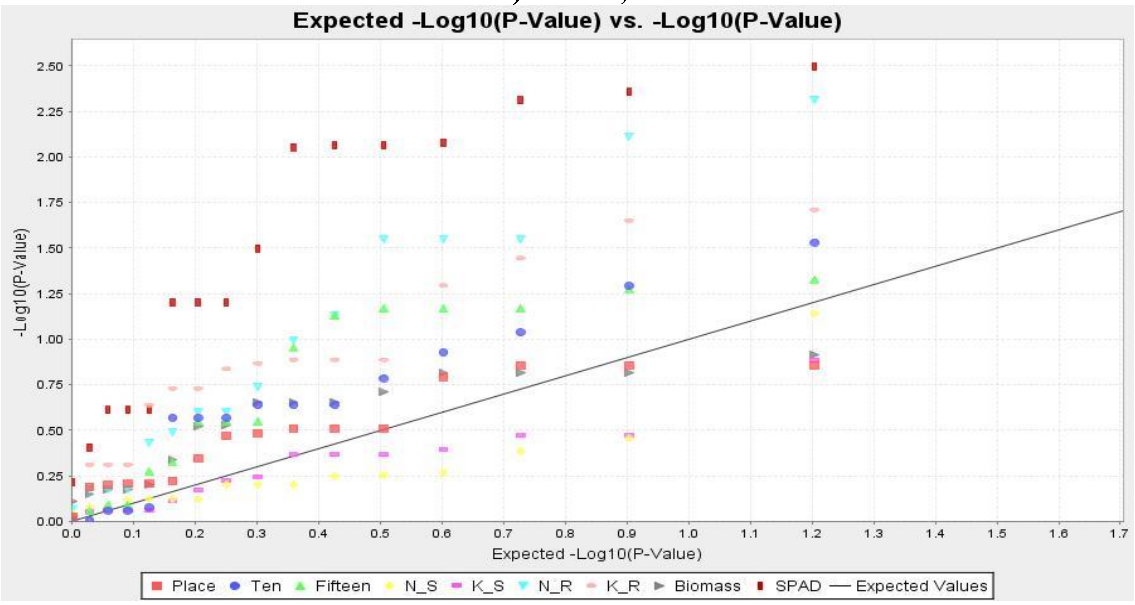

c) HKT1;3

(Continued from page 3)

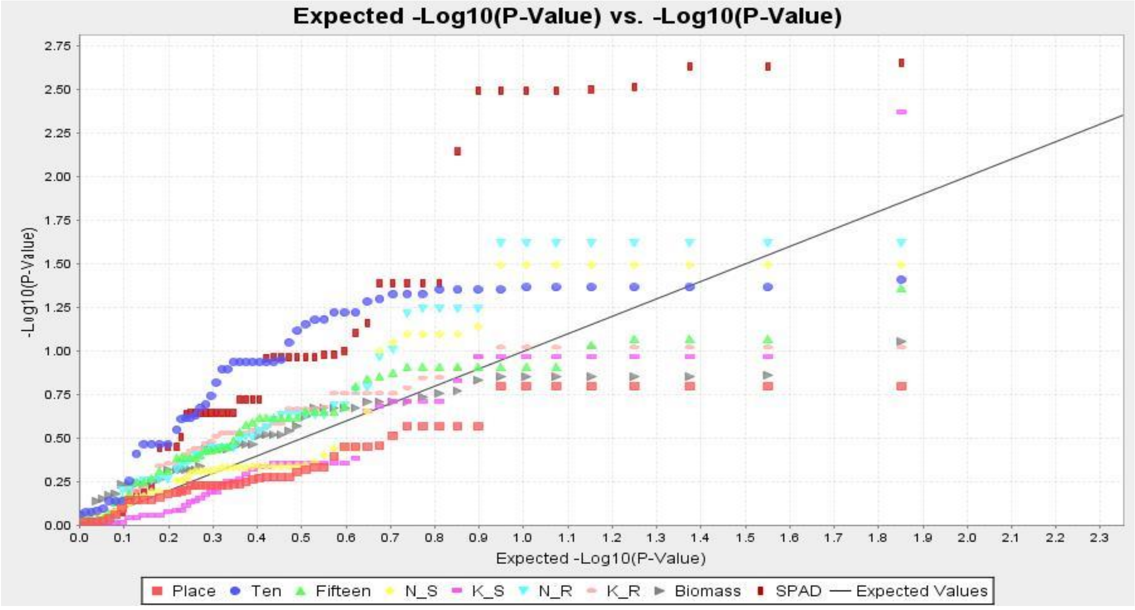

d) HKT1;4

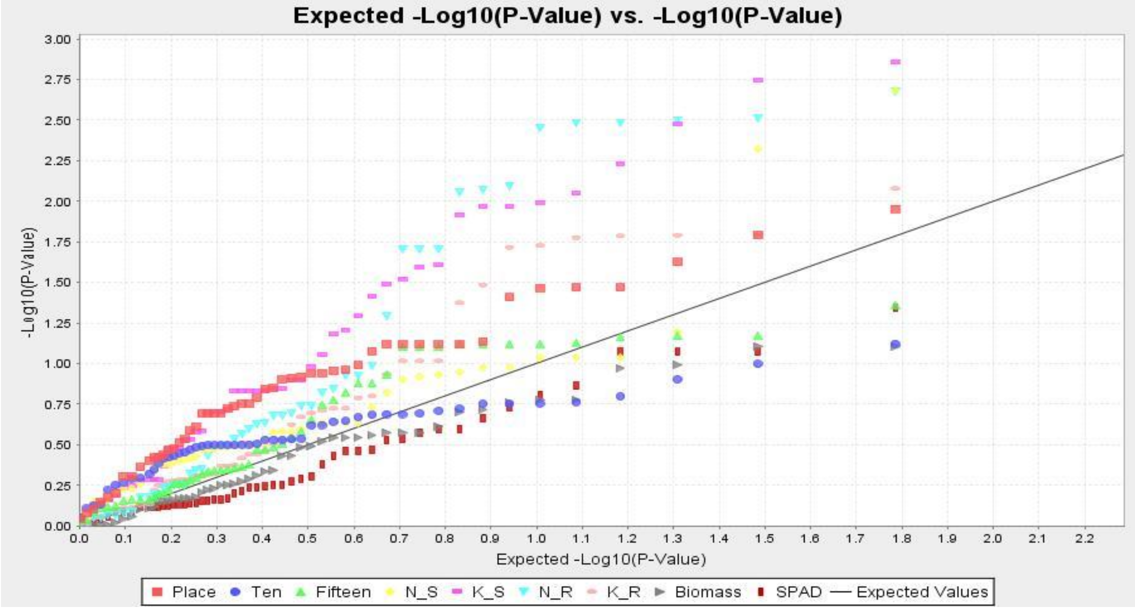

e) HKT1;5

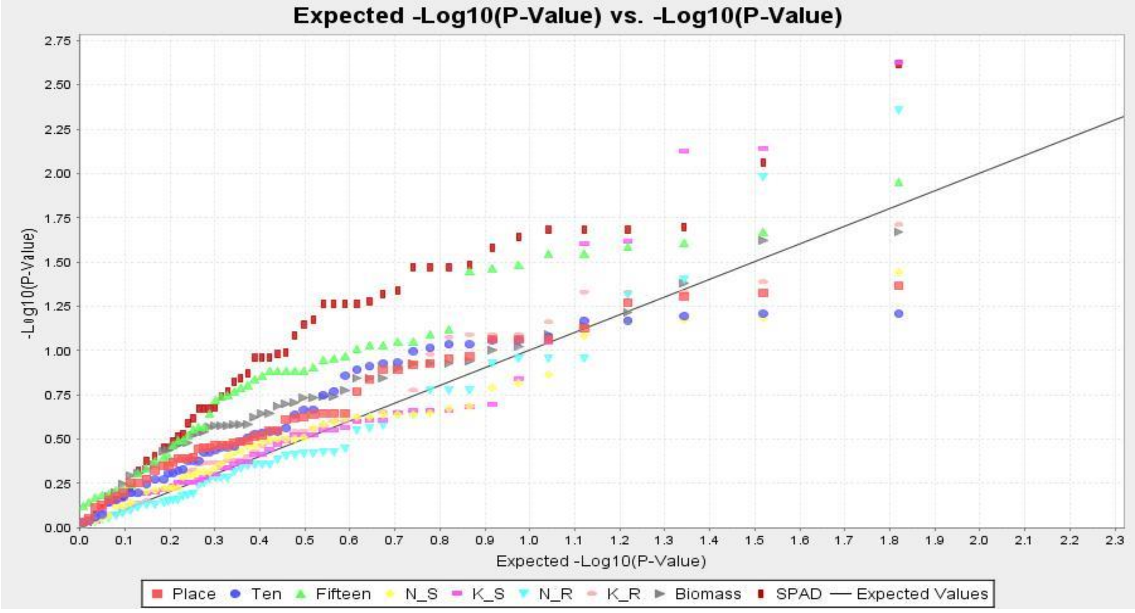

f) HKT2;1

(Continued from page 4)

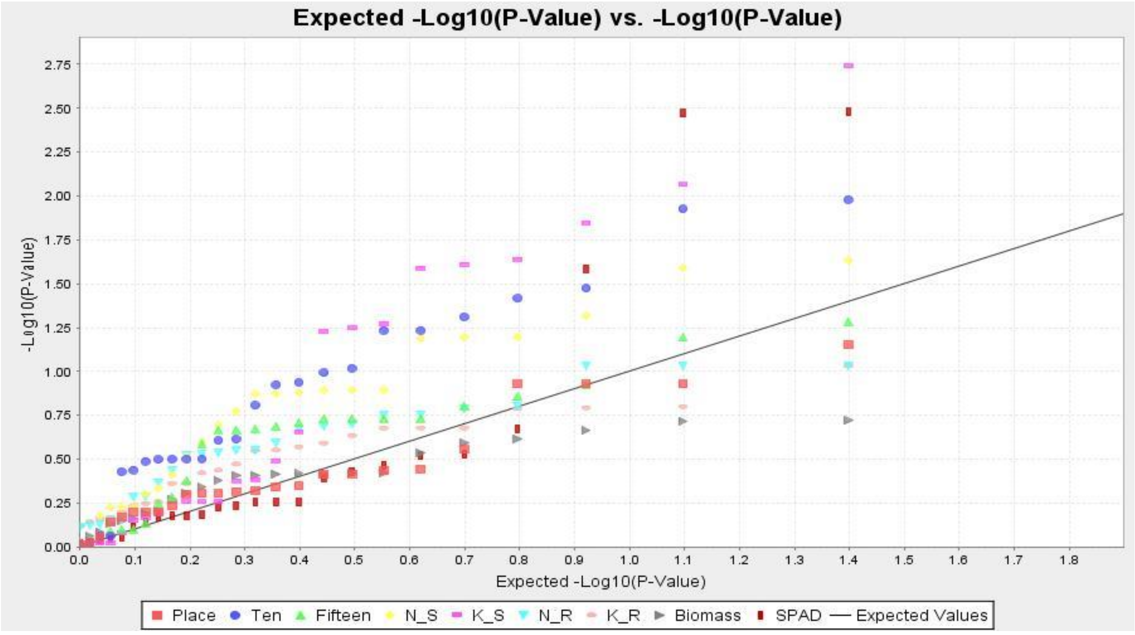

g) HKT2;3

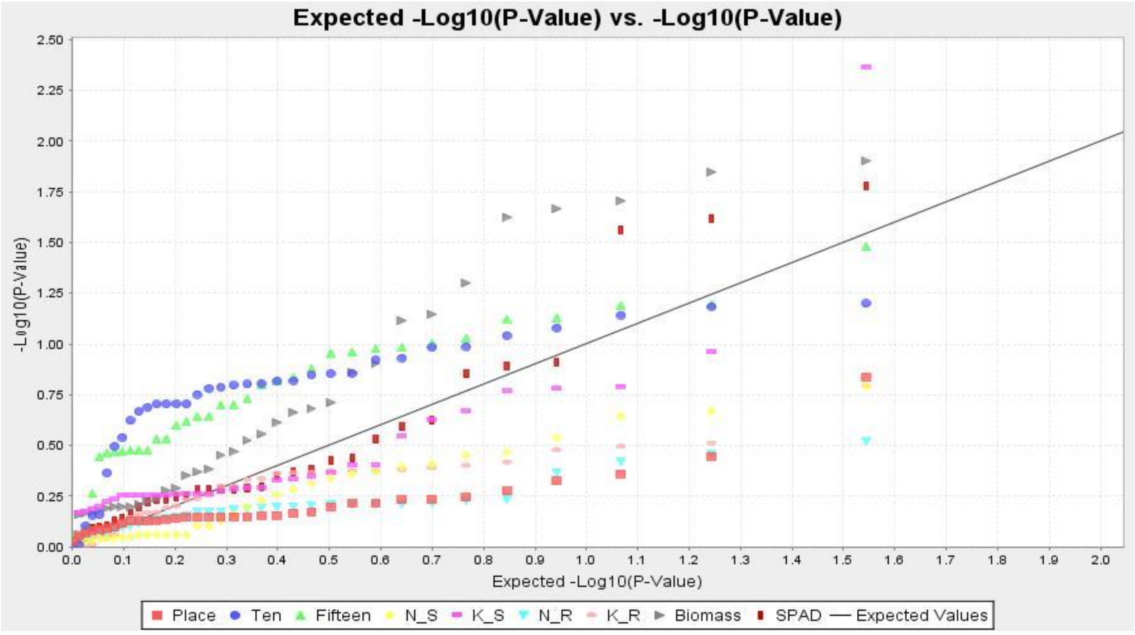

h) HKT2;4

Supplementary Figure 2: Q-Q plots of HKT genes obtained after MLM based association of SNP with traits
